# A Potent Inhibitor of Phosphoinositide 3-Kinase (PI3K) and Mitogen Activated Protein (MAP) Kinase Signalling, Quercetin (3, 3', 4', 5, 7-Pentahydroxyflavone) Promotes Cell Death in Ultraviolet (UV)-B-Irradiated B16F10 Melanoma Cells

**DOI:** 10.1371/journal.pone.0131253

**Published:** 2015-07-06

**Authors:** Rather A. Rafiq, Afnan Quadri, Lone A. Nazir, Kaiser Peerzada, Bashir A. Ganai, Sheikh A. Tasduq

**Affiliations:** 1 PK-PD and Toxicology Division, CSIR-Indian Institute of Integrative Medicine, Canal Road, Jammu Tawi, Jammu and Kashmir, India; 2 Academy of Scientific and Innovative Research (AcSIR), New Delhi, India; 3 Centre of Research for Development (CORD), University of Kashmir, Srinagar, Jammu and Kashmir, India; Taipei Medical University, TAIWAN

## Abstract

Ultraviolet (UV) radiation–induced skin damage contributes strongly to the formation of melanoma, a highly lethal form of skin cancer. Quercetin (Qu), the most widely consumed dietary bioflavonoid and well known inhibitor of phosphoinositide 3-kinase (PI3K) and mitogen activated protein (MAP) kinase signalling, has been reported to be chemopreventive in several forms of non-melanoma skin cancers. Here, we report that the treatment of ultraviolet (UV)-B-irradiated B16F10 melanoma cells with quercetin resulted in a dose dependent reduction in cell viability and increased apoptosis. The present study has brought out that the pro-apoptotic effects of quercetin in UVB-irradiated B16F10 cells are mediated through the elevation of intracellular reactive oxygen species (ROS) formation, calcium homeostasis imbalance, modulation of anti-oxidant defence response and depolarization of mitochondrial membrane potential (ΔΨ_M_). Promotion of UVB-induced cell death by quercetin was further revealed by cleavage of chromosomal DNA, caspase activation, poly (ADP) ribose polymerase (PARP) cleavage, and an increase in sub-G1 cells. Quercetin markedly attenuated MEK-ERK signalling, influenced PI3K/Akt pathway, and potentially enhanced the UVB-induced NF-κB nuclear translocation. Furthermore, combined UVB and quercetin treatment decreased the ratio of Bcl-2 to that of Bax, and upregulated the expression of Bim and apoptosis inducing factor (AIF). Overall, these results suggest the possibility of using quercetin in combination with UVB as a possible treatment option for melanoma in future.

## Introduction

Melanoma arises from the malignant transformation of melanocytes, the pigment producing cells of skin. Melanoma represents only 5% of all the different forms of skin cancers, yet they account for the vast majority of skin cancer related deaths (~75%) [1, 2]. Therefore, effective prevention of melanoma is urgently needed.

Human skin is directly and continuously exposed to solar ultraviolet (UV) radiations. UV radiation generates a range of biological effects in the skin, which includes premature skin aging, immunosuppression, inflammation, cancer, and cell death [3, 4]. Skin cells respond to UV exposure in a variety of ways ranging from activation of pathways that promote survival to eliciting programmed cell death that eliminates altered cells [5]. Whether a cell lives or fails in response to UV exposure is often determined by proliferative efficiency, DNA repair capacity, and the ability to induce proteins that either promote or inhibit the cell death process. Ultraviolet radiation, in particular UVB (λ, 290–320 nm) is known to alter cellular functions via DNA damage, activation of death receptors, depletion of anti-oxidant defences, generation of reactive oxygen species (ROS), and the resultant alterations in a large variety of signalling events [6]. The UVB-induced ROS are usually thought to cause oxidative stress and subsequent damage to membrane lipids, proteins and DNA [7]. To mitigate ROS mediated oxidative damage, living cells have acquired various defense systems including non-enzymatic (α-D-tocopherol, ascorbate) and enzymatic antioxidants (catalase, Cu/Zn SOD) [8, 9]. Nuclear factor erythroid 2–related factor 2 (Nrf-2) is a nuclear transcription factor that in response to oxidative stress regulates coordinated induction of an array of cytoprotective gene expression leading to cellular protection [10, 11]. It has been recognised that UVB-induced cell death occurs through the depolarisation of mitochondrial membrane potential (ΔΨ_M_) and release of pro-apoptotic triggers such as cytochrome c and apoptosis inducing factor (AIF) [6]. Further, proteins of Bcl-2 family constitute a critical control point in regulating mitochondrial membrane permeabilization in response to many types of exogenous stressors [12]. Besides, the regulation of cell cycle progression and apoptotic response is crucial for maintaining cellular homeostasis [13]. UVB is known to induce a G1 block in human HaCaT keratinocytes, human melanocytes, Cloudman melanoma cells, and to affect S phase progression [14]. Furthermore, NF-κB plays a crucial role in the maintenance of skin homeostasis and regulation of cell survival, proliferation and apoptosis resistance [15]. Other signalling pathways documented to play an important role in the response of cells to UVB-irradiation include Ras-Raf-MEK-ERK pathway and phosphatidylinositol-3-kinase (PI3K)/Akt survival signals. In addition to these signalling molecules, C-Jun N-terminal kinase (JNK) and p-38 subgroups of mitogen-activated protein kinases have been suggested to play critical role in apoptosis, cell proliferation, and/or differentiation [16, 17].

**Fig 1:**
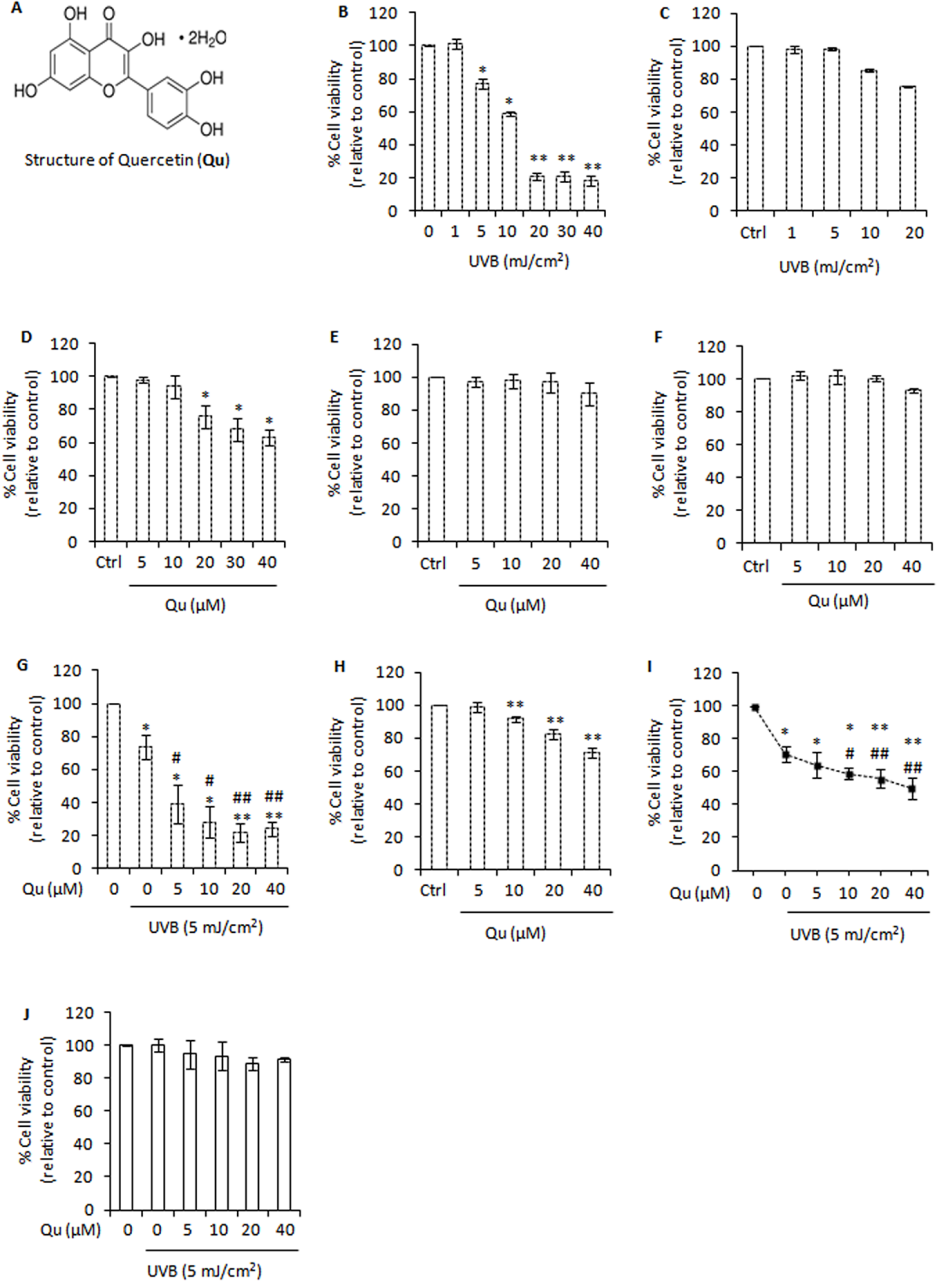
Quercetin promotes UVB-induced cell death. A, structure of quercetin (**Qu**). B, analysis of cell viability using the MTT assay in B16F10 cells at 24 h post-UVB irradiation. *Columns*, mean of three experiments; *bars*, SD. *, P<0.05; **, P<0.01 for control versus treated. C, analysis of cell viability in Hs68 human fibroblast cells at 24 h post-UVB irradiation. D, analysis of cell viability using the MTT assay in B16F10 cells treated with Qu. *, P<0.05; **, P<0.01 for control versus Qu treated. E, analysis of cell viability using the MTT assay in human HaCaT keratinocytes at 24 h post-Qu treatment. F, analysis of cell viability using the MTT assay in Hs68 human fibroblast cells at 24 h post-Qu treatment. G, analysis of cell viability using the MTT assay in B16F10 cells treated with Qu and/or UVB (5 mJ/cm^2^) for 24 hours. *, P<0.05; **, P<0.01 for control versus treated; #, P<0.05; ##, P<0.01 for UVB-alone treatment versus UVB + Qu treatments. H, analysis of cell viability using the MTT assay in A375 human melanoma cells at 24 h post-Qu treatment. I, analysis of cell viability using the MTT assay in A375 cells treated with Qu and/or UVB (5 mJ/cm^2^) for 24 hours. J, analysis of cell viability in Hs68 cells treated with Qu and/or UVB for 24 hours.

Quercetin (3, 3', 4', 5, 7-pentahydroxyflavone, Fig 1A) is a diphenyl propanoid widely distributed in fruits and vegetables, with an average daily intake of 25–30 mg [18]. Quercetin displays antioxidant, anti-inflammatory, antimetastatic and anticancer activities [19–22]. Further, quercetin shows potent anti-melanoma activity and strongly inhibited murine B16F10 cells lung metastasis in an animal model [23, 24].

Exposure of skin to UVB generally leads to the formation of cyclobutane pyrimidine dimers (CPDs), pyrimidine (6–4) pyrimidone photoproducts (6-4PPs) and their dewar valence isomers [25]. The UVB–induced lethal or potentially lethal damage to the DNA of cell initiates cellular recovery mechanisms, which involve activation of DNA damage response pathways, cell cycle arrest and apoptosis. However, some DNA damaged cells evade apoptosis, lose mitotic and differentiation control and eventually become cancerous cells [6]. Therefore, new strategies for induction of apoptosis and/or other cell death mechanisms, such as sub-G1 cell cycle arrest or necrosis deserve examination. Here we have used B16F10 cells as an *in vitro* melanoma model to understand the mechanistic basis for the pro-apoptotic effects of quercetin in UVB–irradiated melanoma cells. It was found that quercetin accelerates cell death in UVB–irradiated B16F10 cells, suggesting the usefulness of this compound as a potent photosensitive agent against the UVB–induced skin damage and skin cancer.

## Materials and Methods

### Reagents

Dulbecco’s modified Eagle’s media (DMEM), Dulbecco’s phosphate buffer saline (DPBS), fetal bovine serum (FBS), penicillin–streptomycin, quercetin dihydrate (Qu), 3-(4,5-dimetylthiazol-yl)-diphenyl tetrazolium bromide (MTT), propidium iodide, rhodamine 123, 2,7-dichlorodihydrofluoresceindiacetate (H_2_DCF-DA), Fluo-3 AM, proteinase K, ribonuclease A, 1,2-bis-(O-aminophenoxy)-ethane-N,N,N’,N’-tetraacetic acid, tetraacetoxymethyl ester (BAPTA-AM), β-nicotinamide adenine dinucleotide reduced disodium salt (β-NADH), ascorbic acid, agarose, dimethyl sulfoxide (DMSO) and anti-β-actin antibody were purchased from Sigma–Aldrich Chemicals (St. Louis, MO). Antibodies against Bcl-2, Bax, Bim, AIF, NF-κB, caspase-3/caspase-8, phospho-MEK, MEK1/2, phospho-ERK, ERK 1/2, phospho-p38, p-38, phospho-JNK, JNK, Nrf-2, catalase, Cu/Zn SOD, PI3K, phospho-Akt, Akt and GAPDH were purchased from Santa Cruz Biotechnology (Santa Cruz Biotechnology, Inc,). FITC Annexin V was purchased from BD Pharmingen. Histone H3 antibody was purchased from Cell Signaling Technology.

Stock solution of quercetin (Qu) was prepared in dimethyl sulfoxide (DMSO) and diluted to the desired final concentration with culture medium just before use. The final DMSO concentration did not exceed 0.1% (v/v).

### Cell Culture

Murine melanoma cell line, B16F10, human foreskin fibroblast cell line, Hs68, human keratinocyte cell line, HaCaT, and human melanoma cell line, A375 were purchased from American Type Culture Collection (Rockville, MD, USA). These cells were maintained in a monolayer culture in 95% air/5% CO_2_ at 37°C in Dulbecco’s modified Eagle’s medium supplemented with FBS (10%), penicillin G (0.012%), streptomycin (0.027%), sodium pyruvate (0.022%) and sodium bicarbonate (0.26%). The percentage of FBS was reduced to 5% for most of the experiments, except otherwise specified.

### UVB Irradiation

UVB irradiation was performed using Daavlin, UVA/UVB Research Irradiation Unit (Bryan, OH, USA) that emits an energy spectrum with high fluence in the UVB (λ, 290–320 nm) range and a peak at 314 nm [26]. UVB irradiation was performed in culture dishes containing a thin layer of warm Dulbecco’s PBS. Control cells were not exposed to UVB.

### MTT Cell Viability Assay

The general viability of cells was measured using MTT assay [26]. The percentage cell viability was calculated as: Cell viability = OD_(treated) /_ OD_(control)_ x 100

### Annexin V/Propidium Iodide Staining and Lactate Dehydrogenase Leakage Assay

Annexin V/Propidium iodide staining was performed as described previously [27]. Briefly, the cell pellet was resuspended in Annexin binding buffer [10 mM HEPES (pH 7.4), 140 mM NaCl, and 2.5 mM CaCl_2_] at a density of 10^6^ cells/mL and incubated for 15 min with FITC-Annexin V and propidium iodide (PI). Analysis was performed on BD FACS Calibur Aria.

Lactate dehydrogenase (LDH) leakage assay was performed as described previously [28]. B16F10 cells were co-treated with UVB (5 mJ/cm^2^) and quercetin and cultured in serum free DMEM for 24 hours. LDH activity was measured in the culture supernatant (130 μL) using 0.2 mM β-NADH and 0.4 mM pyruvic acid upto 200 μL Dulbecco’s PBS (pH 7.4). LDH activity was proportional to the rate of NADH oxidation measured by the absorbance at 334 nm (OD/min) using a microplate reader (Multiskan Spectrum; Thermo Electron Corporation). LDH activity in the culture supernatant was expressed as percentage fold change relative to control.

### Analysis of Sub-G1 Apoptotic Cells and DNA Fragmentation Assays

Analysis of sub-G1 cells was carried out as described previously [26]. Briefly, B16F10 cells were harvested and fixed in ice-cold 70% ethanol at -20°C overnight. Fixed cells were centrifuged and incubated in FACS staining solution (50 μg/mL of propidium iodide in Dulbecco’s PBS containing 10 μg/mL DNase free RNase A) at 37°C for 30 minutes. The fluorescence intensity of propidium iodide (PI) was measured using BD FACS Calibur Aria.

DNA fragmentation analysis was performed as described previously [29]. Both adherent and floating cells were harvested and washed in ice-cold Dulbecco’s PBS containing 10 mM EDTA. The cell pellet was solubilized in lysis buffer [10 mM Tris-HCl (pH 8.0), 100 mM NaCl, 5 mM EDTA, 5% triton X-100, 0.25% SDS, 400 μg/mL RNase A] and incubated at 37°C for 90 minutes. After incubation, cell pellet was treated with proteinase K (200 μg/mL) at 50°C for further 1 hour. DNA was extracted with phenol-chloroform-isoamyl alcohol (25:24:1) and analysed electrophoretically on 1.8% agarose gel containing 0.1 μg/mL of ethidium bromide.

### Determination of Mitochondria Membrane Potential (ΔΨ_M_)

Mitochondrial membrane potential (ΔΨ_M_) dissipation was measured after staining of cells with rhodamine 123 (5 μM) for 15 minutes at 37°C. Both adherent cells and floating cells were collected, centrifuged and washed with Dulbecco’s PBS. The intensity of fluorescence was measured in FL-1 channel by BD FACS Calibur Aria.

### Determination of Intracellular ROS and Calcium Signals

Generation of reactive oxygen species (ROS) was measured as described previously [30]. B16F10 cells were treated with quercetin for 24 hours and subsequently irradiated with 5 mJ/cm^2^ of UVB. Soon after irradiation, cells were incubated in 5 μM H_2_DCFDA reagent for 20 minutes at 37°C. The generation of ROS was measured by the change in fluorescence due to the production of 2',7'-dichlorofluorescein (DCF) by BD FACS Calibur Aria.

The intracellular free calcium was measured as described previously [31]. Briefly, the cells were collected, washed twice with ice-cold calcium and magnesium-free Dulbecco’s PBS and then incubated with Fluo-3 AM (5 μM) for 15 min at 37°C in dark. Analysis was performed on BD FCAS Calibur Aria.

To determine whether the changes in intracellular Ca^2+^ are induced due to UVB–induced ROS generation, B16F10 cells were pre-treated with quercetin for 24 hours and supplemented with ascorbic acid (1 mM) for 1 hour before UVB irradiation. Following UVB irradiation, B16F10 cells were loaded with Fluo-3 AM (5 μM) for 15 minutes in dark and analyzed on confocal laser scan microscope (Olympus Fluoview FV1000). Quantitative analysis of intracellular Ca^2+^ was performed using the Fluoview FV1000 software.

### Preparation of Cell Lysate and Western Blotting

Whole cell lysate was prepared by solubilizing cell pellet in RIPA buffer (Sigma; R-0278) over ice for 40 minutes as described previously [32]. The lysate was cleared by centrifugation at 14000 g for 15 min at 4°C and the supernatant was collected as whole cell lysate. Cytosolic and nuclear fractions were prepared as described previously with minor modifications [33]. Protein estimation was done by Bradford’s method.

Western blotting was performed using equal amounts of protein, including pre-stained molecular weight markers (Precision Plus Protein Standards Kaleidoscope, Bio Rad). The proteins were electrotransferred to polyvinylidene difluoride (PVDF) membranes (Immobilon P 0.45 μm, Millipore). After blocking for 2 h at room temperature in 5% fat-free milk powder or 3% BSA in TBST (Tris-buffered saline containing 0.05% Tween 20, pH 8.0), membranes were incubated overnight with specific antibodies. After 30 minutes washing with TBST, membranes were probed with different HRP-conjugated secondary antibodies. Detection of specific proteins was carried out by Immobilon Western (Chemiluminescent HRP substrate, Millipore) mediated chemiluminescence by ChemiDoc XRS+ (Bio Rad). Densitometry of specific bands was done using Image Lab Software version 3 (Bio Rad).

### Statistical Analysis

Numerical data were presented as mean ± standard deviation (SD). Statistical analysis of numerical data between control versus treatments (*) and UVB-alone treatment versus quercetin and UVB treatment (#) were performed by applying Student’s t-test. A P value <0.05 was considered as statistically significant.

## Results

### Quercetin Shows Potent Growth Inhibition of UVB-irradiated B16F10 Cells

To determine the effect of UVB irradiation on cell viability, B16F10 cells were exposed to UVB (1–40 mJ/cm^2^) and cell viability was measured at 24 h post-UVB irradiation. Our data showed that UVB exposure resulted in a significant decrease in the viability of B16F10 cells (Fig 1B). Upon UVB irradiation at 5 mJ/cm^2^, the viability of B16F10 cells decreased by around 22% relative to un-irradiated control cells (Fig 1B). However, UVB (5 mJ/cm^2^) induced no significant cell death in normal Hs68 human fibroblast cells, and was therefore selected for further experiments (Fig 1C). Next B16F10 cells were treated with quercetin (5–40 μM) and cell viability was analyzed. Quercetin (5 μM) induced no cell death in B16F10 cells, whereas 10, 20, 30, and 40 μM quercetin decreased the cell viability by 6%, 24%, 32% and 37% respectively (Fig 1D). To show the selectivity or specificity in the pro-apoptotic effects of quercetin, human HaCaT keratinocytes and Hs68 fibroblast cells were treated with quercetin and cell viability was analyzed. Although quercetin (5–20 μM) induced no significant cell death in HaCaT and Hs68 cells, limited growth inhibition was recorded at 40 μM (Fig 1E and 1F). These results suggest that the effectiveness of quercetin to induce cell death is cell type specific and depends on the dose of administration. Next, we investigated whether quercetin promotes cell death in UVB–irradiated B16F10 cells. We found that the treatment of UVB-irradiated B16F10 cells with 5, 10, 20, and 40 μM quercetin increased the percentage of cell death to around 61%, 65%, 72% and 74% respectively (Fig 1G). We also studied the key effects of quercetin in UVB–irradiated A375 human melanoma cells. Our results showed that quercetin induces growth inhibition (Fig 1H) and efficaciously enhanced the UVB–induced cell death in A375 human melanoma cells (Fig 1I). Unlike melanoma cells, quercetin failed to promote cell death in UVB-irradiated non-tumorigenic Hs68 fibroblast cells (Fig 1J). Overall, these results indicated that quercetin is capable of eliciting different responses that need to be well characterized to fully understand how this compound might be useful in preventing or treating skin cancer.

### Combined UVB and Quercetin Treatment Enhances Apoptotic Cell Death and Induces Caspase Activation and PARP-1 Cleavage

**Fig 2:**
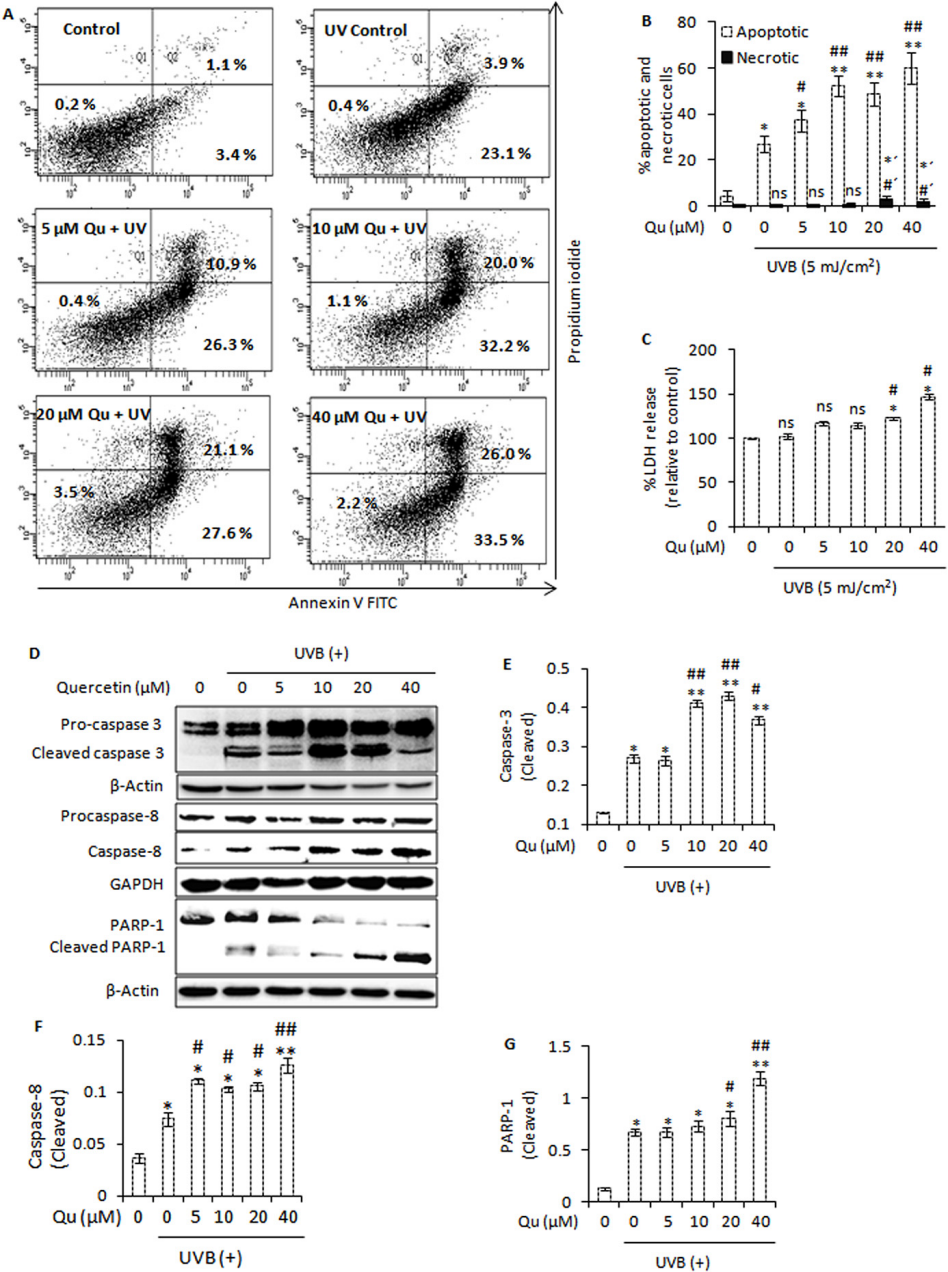
Combined UVB and quercetin treatment enhances apoptotic cell death and induces caspase activation and PARP-1 cleavage. A, Annexin V/propidium iodide assay followed by flow cytometric analysis of apoptosis and necrosis in B16F10 cells at 24 h post-UVB/Qu treatment. B, represents the percentage of apoptotic and necrotic cells. *, P<0.05; **, P<0.01 for control (apoptotic) versus treated; #, P<0.05; ##, P<0.01 for UVB-alone treatment (apoptotic) versus UVB + Qu treatments; *´, P< 0.05 for control (necrotic) versus treated; #', P<0.05 for UVB-alone (necrotic) versus UVB + Qu treatments. C, analysis of lactate dehydrogenase (LDH) leakage in cells treated with Qu and/or UVB. *, P<0.05 for control versus treated; #, P<0.05 for UVB-alone treatment versus UVB + Qu treatment; ns, statistically not significant. D, western blot analysis of caspase-3/caspase-8 activation and PARP-1 cleavage in B16F10 cells at 24 h post-UVB/Qu treatment. The signals for the cleaved forms of caspase-3 (E), caspase-8 (F) and PARP-1 protein (G) were quantified and normalized against β-actin, GAPDH and β-actin respectively using Image Lab software Version 3.0 (BioRad).

To determine whether the decrease in cell viability is caused by enhanced apoptosis, we performed Annexin V/propidium iodide double staining to compare apoptosis and necrosis between control and treated cells. In agreement with the above results, we found that quercetin caused a substantial increase in UVB-induced apoptotic response, with nearly 26%, 32%, 27% and 33% of cells found to be in the early phase of apoptosis (Fig 2A and 2B). No significant increase in necrotic cell death was detected with or without UVB or quercetin (5–10 μM) treatment (Fig 2B). However, subtle but significant increase in necrotic cell death was detected in UVB-irradiated B16F10 cells treated with 20 and 40 μM quercetin relative to control necrotic cells. Next we examined the effect of quercetin on lactate dehydrogenase (LDH) activity in UVB-irradiated B16F10 cells. Treatment of UVB-irradiated B16F10 cells with 5, 10, 20, and 40 μM quercetin induced 1.17-, 1.14-, 1.22-, and 1.46-fold increase in LDH activity respectively relative to un-irradiated control cells (Fig 2C). Western blot analysis also revealed that combined UVB and quercetin treatment enhanced activation of caspases and cleavage of poly(ADP-ribose) polymerase (PARP), which is a hallmark feature of apoptosis (Fig 2D–2G).

### Quercetin Enhances the UVB–induced Sub-G1 Cell Cycle Arrest and Cleavage of Chromosomal DNA

**Fig 3:**
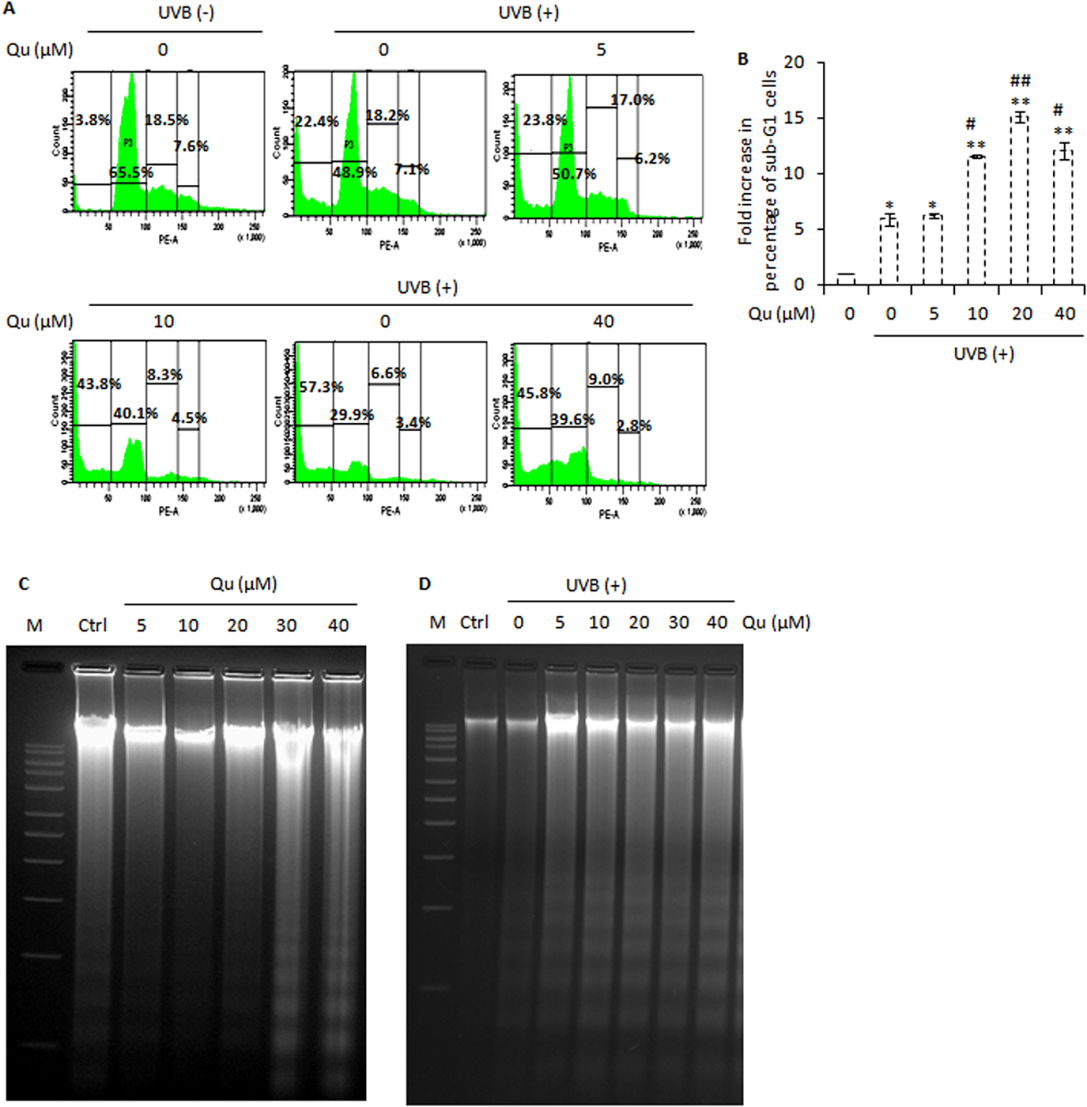
Combined UVB and quercetin treatment enhances sub-G1 cell cycle arrest and cleavage of chromosomal DNA. A, analysis of cell cycle progression in B16F10 cells treated with Qu and/or UVB. Cells were collected (including floating cells), stained with propidium iodide and analyzed for DNA content by BD FACS Calibur Aria. ‘Sub-G1 peak’ contains apoptotic cells. B, represents the fold increase in the percentage of sub-G1 cells relative to control. *, P<0.05; **, P<0.01 for control versus treated; #, P<0.05; ##, P<0.01 for UVB-alone treated versus UVB + Qu treated. C, analysis of DNA fragmentation in B16F10 cells at 24 h post-Qu treatment. D, analysis of DNA fragmentation in B16F10 cells at 24 h post-UVB/Qu treatment.

Here, we analyzed the effect of quercetin on induction of sub-G1 cell cycle arrest in UVB-irradiated B16F10. UVB irradiation increased the percentage of hypodiploid sub-G1cells (22%) relative to control cells (3%). Quercetin markedly increased the UVB-induced sub-G1 cell cycle arrest, with nearly 23%, 43%, 57% and 46% of cells found in the sub-G1 phases of cell cycle (Fig 3A and 3B). Sub-G1 cells are known to arise as a result of cleavage of chromosomal DNA during the late stages of apoptotic cell death [34]. Therefore, we evaluated whether the cytotoxic action of quercetin on UVB–irradiated B16F10 cells was associated with cleavage of chromosomal DNA which is a hallmark feature of apoptosis [34]. We first treated the B16F10 cells with quercetin and analyzed the cleavage of chromosomal DNA at 24 h post- quercetin treatment. It was found that quercetin caused no or minimal cleavage of chromosomal DNA upto a concentration of 20 μM. However, quercetin at higher concentrations (30–40 μM) induced detectable and significant DNA fragmentation in B16F10 cells (Fig 3C). Upon UVB irradiation at 5 mJ/cm^2^, cleavage of chromosomal DNA was detected in B16F10 cells. Interestingly, combined UVB and quercetin treatment induced marked and substantial DNA fragmentation (Fig 3D). Taken together, these results indicated that quercetin causes B16F10 cells to undergo apoptosis as a result of UVB–induced DNA fragmentation.

### Combined UVB and Quercetin Treatment Induces Mitochondria Dysfunction and Modulates the Expression of various Pro- and Anti-apoptotic Proteins

**Fig 4:**
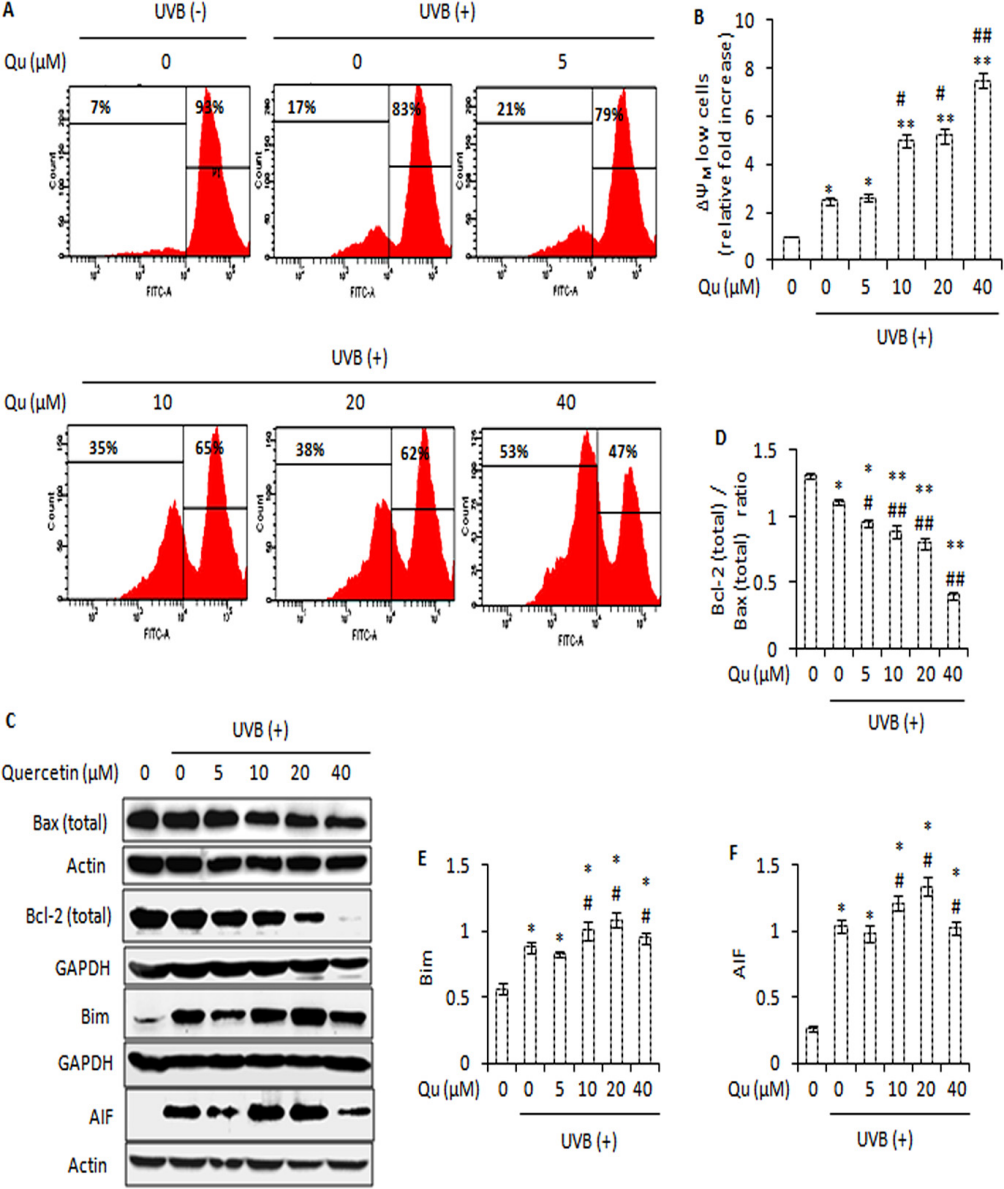
Quercetin promotes UVB-induced mitochondrial membrane potential (ΔΨ_M_) dissipation and modulates the expression of various pro- and anti-apoptotic proteins. A, analysis of mitochondrial membrane potential (ΔΨ_**M**_) in B16F10 cells treated with Qu and/or UVB. B, represents the fold increase in the percentage of ΔΨm low cells relative to control cells. C, immunoblot analysis of Bax, Bcl-2, Bim and AIF in B16F10 cells treated with Qu and/or UVB. D, signals were quantified for Bax and Bcl-2 and expressed as Bcl-2 (total)/Bax (total) ratio for each treatment. E and F, represents the densitometric analysis of Bim and AIF respectively. *, P<0.05; **, P<0.01 for control versus treatments; #, P<0.05, ##, P<0.01 for control versus UVB-alone treatment versus UVB + Qu treatments.

In addition to being the cell’s powerhouse, the site of adenosine triphosphate (ATP) synthesis, mitochondria can also be the source of signals that can initiate programmed cell death [35]. The majority of ATP synthesis in living cells is driven by the electrochemical gradient built across the inner mitochondrial membrane known as mitochondrial membrane potential, symbolized as ΔΨ_M_. During apoptosis, the mitochondrial membrane potential (ΔΨ_M_) of a cell falls, facilitating the release of cytochrome c and activation of downstream cellular apoptotic machinery [36]. Here, we have studied the effect of quercetin on the mitochondrial physiology of UVB-irradiated B16F10 cells. Flow cytometry showed that 5 mJ/cm^2^ UVB caused 2.5-fold increase in the percentage of ΔΨ_M_ low cells relative to control. However, the treatment of UVB-irradiated B16F10 cells with 5, 10, 20 and 40 μM quercetin caused 2.6-, 5-, 5.1- and 7.5-fold increase in the percentage of ΔΨ_M_ low cells (Fig 4A and 4B). During UVB irradiation, depolarization of mitochondrial membrane potential (ΔΨ_M_) is regulated by the proteins of Bcl-2 family [12] and the outcome of death signal usually depends on the balance between the positive and negative apoptotic regulators of the Bcl-2 family [37]. UVB irradiation decreased the ratio of Bcl-2 to that of Bax. Interestingly, quercetin caused significant and gradual decrease in the ratio of Bcl-2 to that of Bax, and upregulated the expression of Bim and apoptosis inducing factor (AIF) (Fig 4C–4F). These changes in the protein expression of Bcl-2 family proteins and mitochondria dysfunction may account for the increased apoptosis.

### Combined UVB and Quercetin Treatment Elevates Reactive Oxygen Species (ROS) and Intracellular Calcium Signals and Disrupts NF-κB Signalling

**Fig 5:**
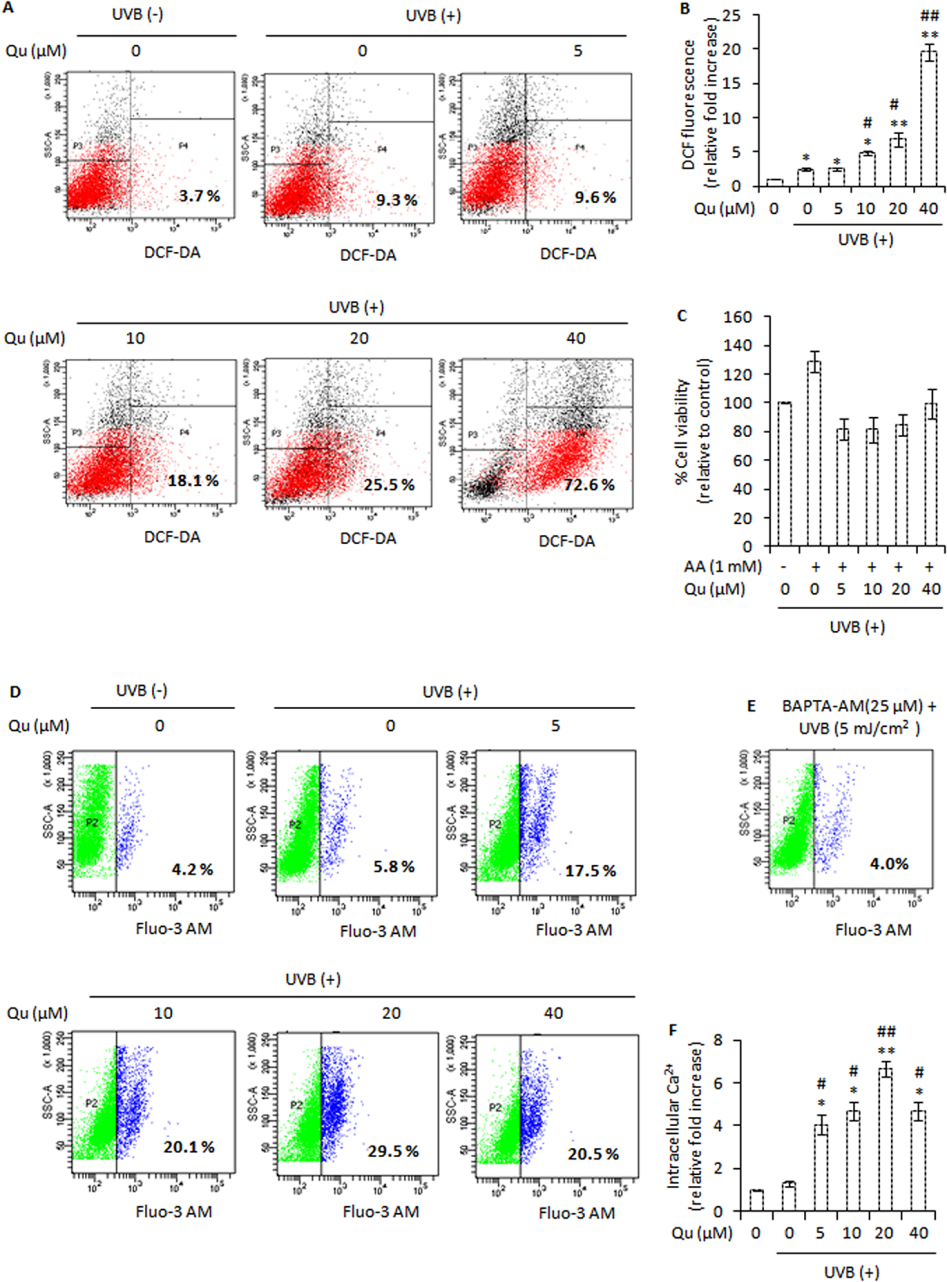
Quercetin elevates UVB-induced reactive oxygen formation and intracellular calcium ion level. A, analysis of reactive oxygen species (ROS) formation immediately after UVB irradiation in B16F10 cells pre-treated with Qu for 24 hours. The generation of ROS was measured using BD FACS Calibur Aria. B, represents the fold increase in DCF fluorescence (a measure of ROS formation) relative to control. *, P<0.05; **, P<0.01 for control versus treatments; #, P<0.05, ##, P<0.01 for control versus UVB-alone treatment versus UVB + Qu treatments. C, effect of ascorbic acid (1 mM) on cell viability in response to treatment of UVB–irradiated B16F10 cells with Qu; UVB control cells were taken as 100% viable. D, analysis of intracellular free Ca^2+^ immediately after UVB exposure in B16F10 cells pre-treated with Qu for 24 hours. E, represents the effect of UVB (5 mJ/cm^2^) on calcium elevation in BAPTA-AM preloaded B16F10 cells. F, represents the fold increase in intracellular free calcium relative to control. *, P<0.05; **, P<0.01 for control versus treatments; #, P<0.05, ##, P<0.01 for control versus UVB-alone treatment versus UVB + Qu treatments.

**Fig 6:**
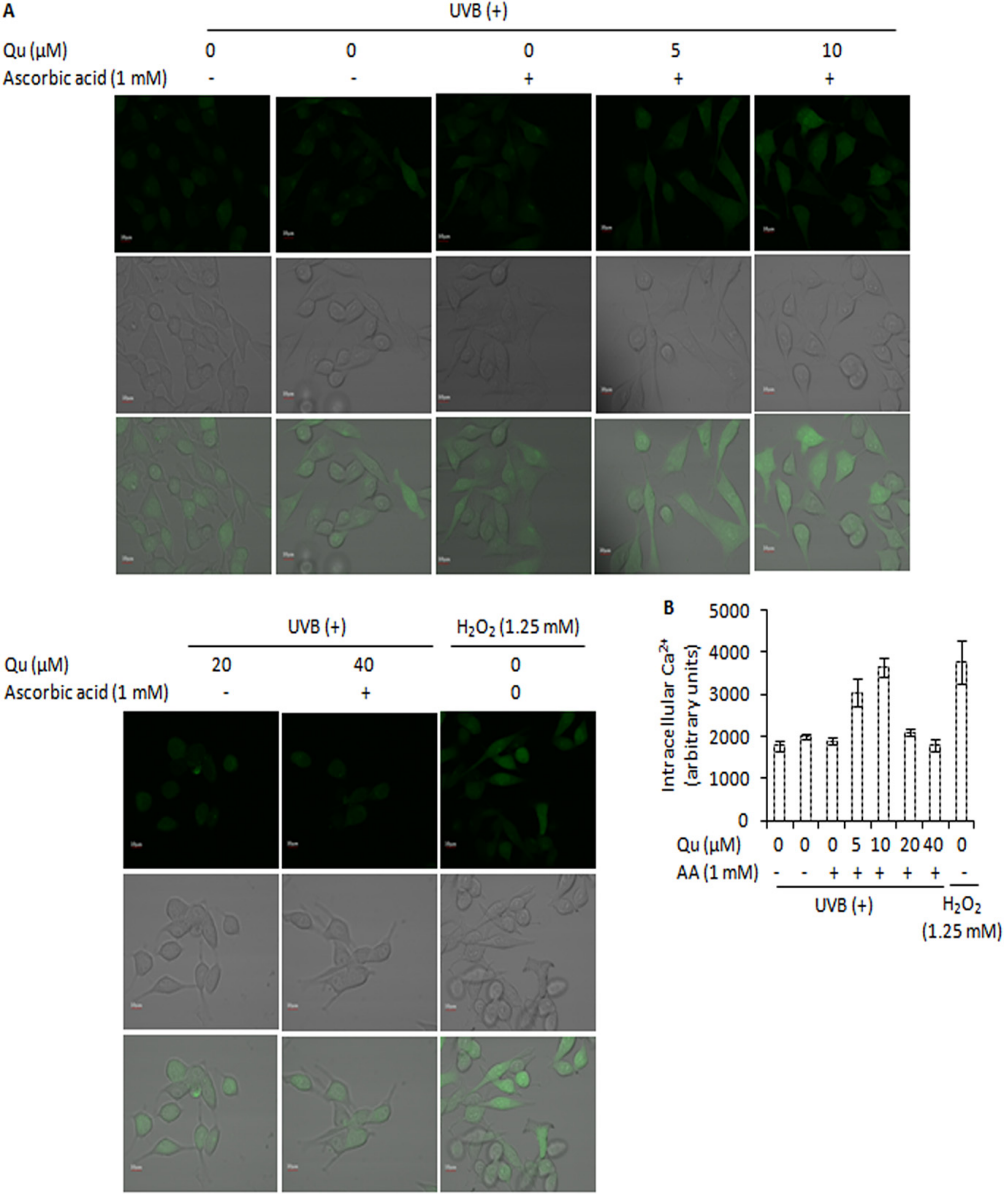
Effect of ascorbic acid (1 mM) on intracellular Ca^2+^ changes in response to treatment of UVB-irradiated B16F10 cells with quercetin. A, B16F10 cells were pre-treated with Qu for 24 h and supplemented with ascorbic acid 1 h before UVB irradiation. Following UVB irradiation, cells were loaded with Fluo-3 AM and analyzed on Olympus Flowview FV1000. B, represents the densitometric measure of the effect of ascorbic acid (AA) on intracellular Ca^2+^ levels in response to treatment of UVB-irradiated B16F10 cells with Qu.

In skin cells, UVB irradiation stimulates the production of reactive oxygen species [38]. In agreement with this, we noted that when B16F10 cells were irradiated with 5 mJ/cm^2^ of UVB, a substantial increase in ROS generation was observed (9.3%; Fig 5A). Next we investigated the contribution of quercetin to UVB-induced ROS generation. UVB irradiation documented 2.5-fold increase in ROS formation. Quercetin actually increased UVB-induced ROS generation. 5, 10, 20, and 40 μM quercetin caused 2.6-, 4.9-, 6.9-, 19.6-fold increase in UVB-induced ROS formation respectively (Fig 5A and 5B). Addition of ascorbic acid (1 mM) 1 h prior and subsequent to co-treatment of UVB and quercetin markedly reduced the pro-apoptotic effects of quercetin in UVB–irradiated B16F10 cells (Fig 5C). These results indicate that this pro-oxidant behaviour of quercetin in UVB–irradiated B16F10 cells could be one of the factors responsible for growth inhibitory effects of quercetin in B16F10 cells. We next studied the effect of quercetin on intracellular free calcium. Changes in intracellular free calcium have been reported to initiate ROS formation in UVB-irradiated human HaCaT keratinocytes [39]. UVB irradiation caused 1.3-fold increase in the percentage of cells with high level of intracellular free calcium (5.8%, p<0.01). However, in UVB-irradiated B16F10 cells, quercetin produced a marked increase in intracellular free calcium. 5, 10, 20 and 40 μM quercetin added subsequent to UVB irradiation caused 4.1-, 4.6-, 6.7-, and 4.7-fold increase in the percentage of cells with high levels of intracellular free calcium (Fig 5D and 5F). Treatment of cells with H_2_O_2_ (1.25 mM) for 10 minutes enhanced the level of intracellular free calcium, indicating that ROS may be responsible for intracellular calcium elevation (Fig 6A and 6B). However, preloading cells with BAPTA-AM, a specific cell-permeable chelating agent of Ca^2+^, decreased the subsequent UVB–induced elevation of intracellular free calcium, indicating that intracellular free calcium elevates in response to UVB irradiation of B16F10 cells (Fig 5E). Blocking ROS using 1 mM ascorbic acid as an anti-oxidant reduced the intracellular calcium changes (Fig 6A and 6B). In addition, ascorbic acid could also reduce the pro-apoptotic effects of quercetin in UVB–irradiated B16F10 cells (Fig 5C). Overall, these results indicated that ROS generated by the combination use of UVB and quercetin may be responsible for the intracellular calcium changes and subsequent cell death.

**Fig 7:**
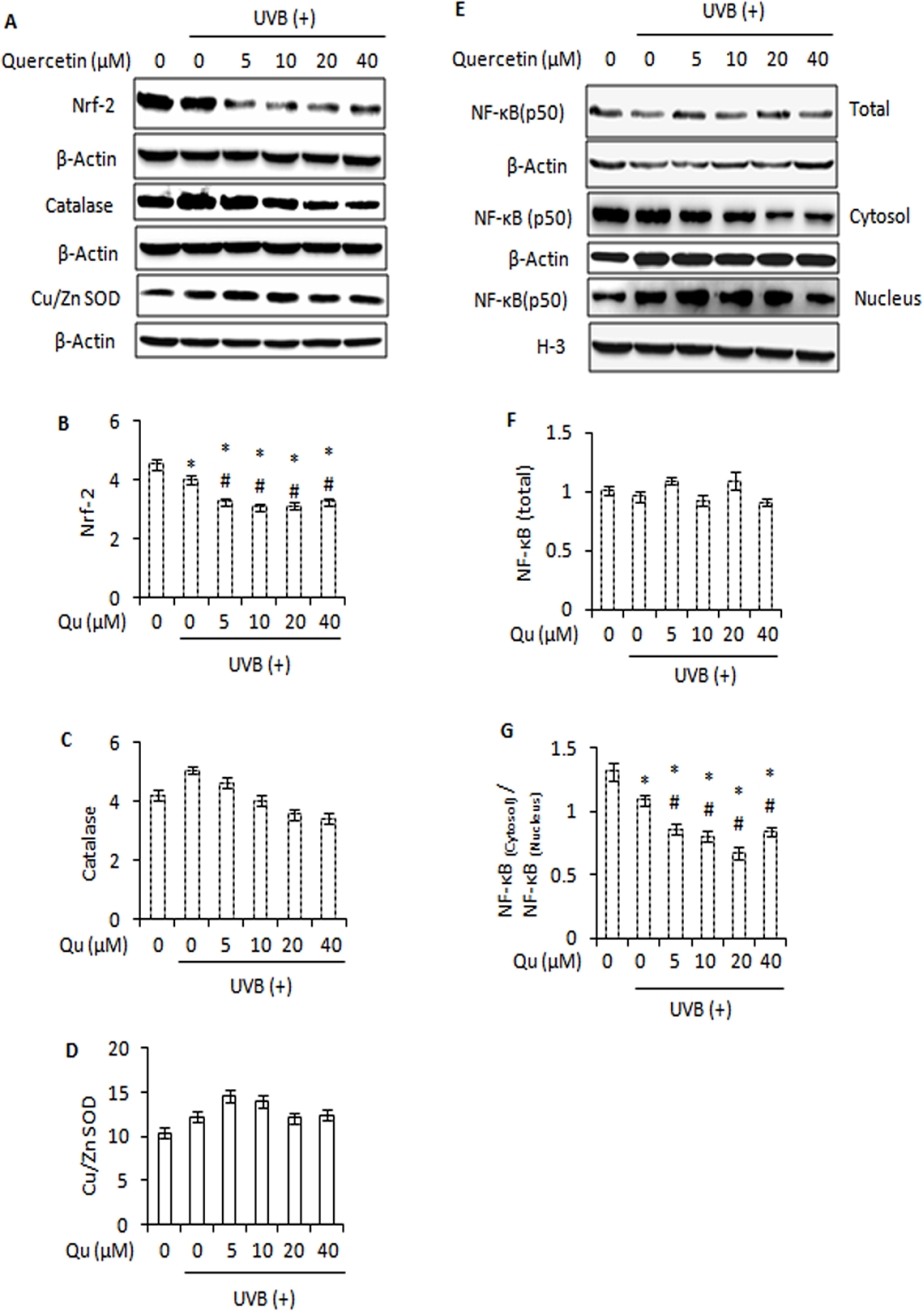
Effect of quercetin on major protein regulators of anti-oxidant defence response in UVB-irradiated B16F10 cells. A, immunoblot analysis of Nrf-2, Catalase and Cu/Zn SOD in B16F10 cells treated with Qu and/or UVB. Signals were quantified for Nrf-2 (B), catalase (C) and Cu/Zn SOD (D) and normalized against β-actin of each band using the Image Lab Software. E, immunoblot analysis of whole cell, cytosolic and nuclear NF-κB in B16F10 cells treated with Qu and/or UVB. F, represents the densitometric analysis of NF-κB (whole cell). G, the signals for cytosolic and nuclear NF-κB were quantified and expressed as ratio of NF-κB_**(cytosol)**_ / NF-κB_**(nucleus)**_ for each treatment.

It has been recognized that exposure of skin to UVB primarily induces the production of superoxide anion radical (O_2_^.-^) and hydrogen peroxide (H_2_O_2_). Nuclear factor erythroid 2-related factor 2 (Nrf-2) is a key transcription factor in the regulation of antioxidant defence response [11]. We observed that UVB irradiation reduced the expression of Nrf-2. After treatment of UVB–irradiated B16F10 cells with quercetin, we observed a further decrease in the expression of Nrf-2 (Fig 7A and 7B). To elucidate the role of catalase, an enzyme that cleaves H_2_O_2_, in response to treatment of UVB–irradiated B16F10 cells with quercetin, we analyzed the expression of catalase by western blotting. It was found that UVB irradiation induced the expression of catalase. In contrast, quercetin decreased the expression of catalase in UVB–irradiated B16F10 cells (Fig 7A and 7C). In addition, UVB irradiation produced an increase in the expression of copper-zinc superoxide dismutase (Cu/Zn SOD), an enzyme that catalyzes the dismutation of superoxide anion radical (O_2_^.-^) to dioxygen (O_2_) and hydrogen peroxide (H_2_O_2_). Although the expression of Cu/Zn SOD protein increased upon treatment of UVB–irradiated B16F10 cells with lower doses of quercetin, a gradually decrease was observed at higher doses of quercetin (Fig 7A and 7D). Decrease in Cu/Zn SOD expression following treatment of UVB-irradiated B16F10 cells with higher doses of quercetin is probably due to over consumption of Cu/Zn SOD in response to excessive superoxide anion radical (O_2_^.-^) formation. Together, these results indicate that alterations in natural anti-oxidant defence system of cells contribute to oxidative damage and cell death.

UVB-induced ROS interacts with NF-κB signalling in many ways. Depending on the cellular context, ROS have various stimulatory or inhibitory roles in NF-κB signalling. NF-κB consists of p50 and p65 (RelA) subunits. Both subunits constitute a larger group of transcription factors: the Rel family [40]. We observed that quercetin caused no further decrease in NF-κB (p50) expression in response to UVB-irradiation (Fig 7E and 7F). However, quercetin markedly enhanced the UVB-induced translocation of NF-κB (p50) from cytosol to nucleus (Fig 7E and 7G). Translocation of NF-κB in response to treatment of UVB-irradiated B16F10 cells with quercetin is in consistence with the role of NF-κB as a cellular redox sensor [41] and mediator of cell death [42].

### Quercetin Attenuates MAPK Signalling and PI3K/Akt Pathway in UVB-irradiated B16F10 Melanoma Cells

**Fig 8:**
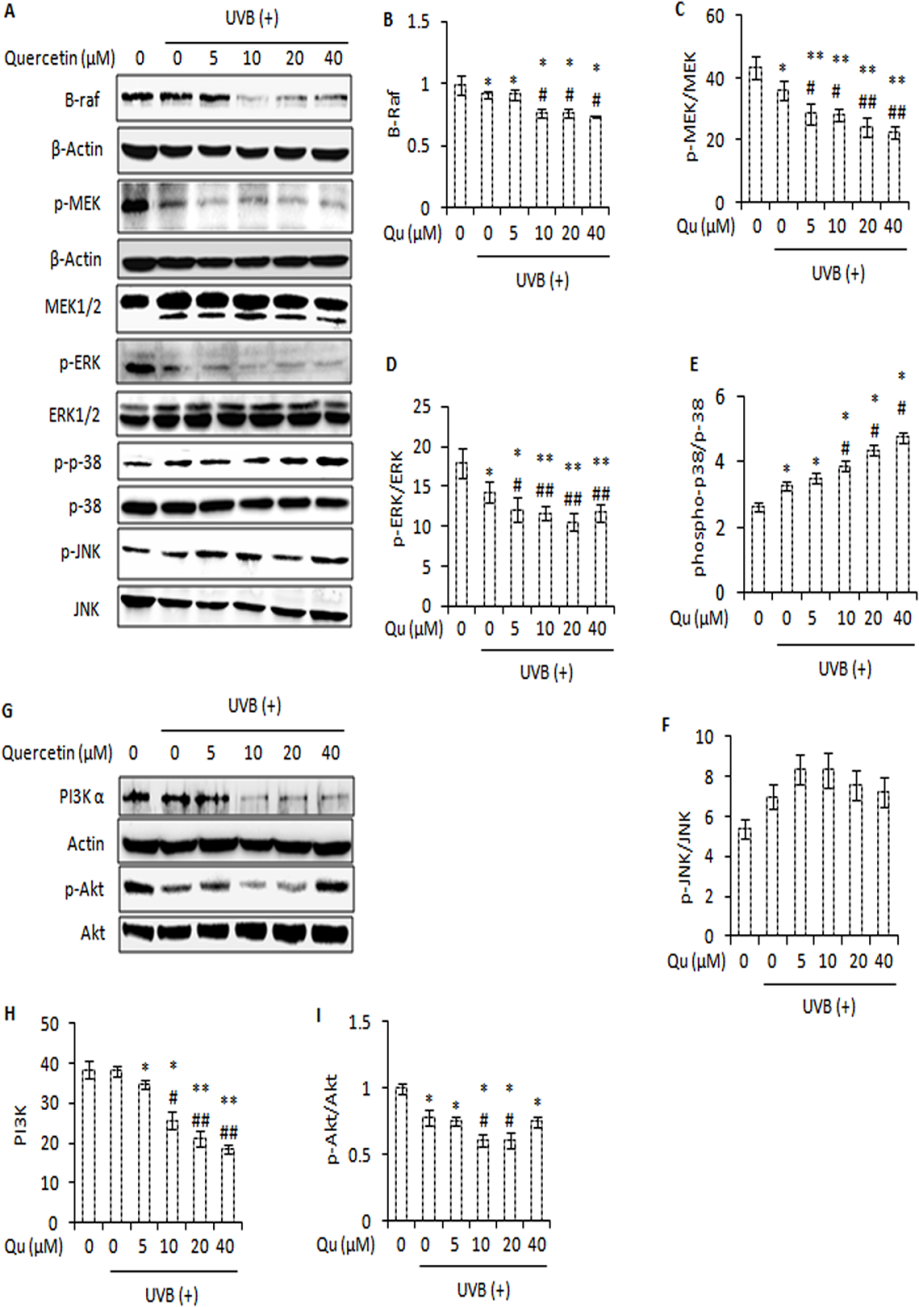
Quercetin attenuates PI3K-Akt pathway and MAPK signalling in UVB-irradiated B16F10 melanoma cells. A, western blot analysis of B-raf, p-MEK, MEK1/2, p-ERK, ERK1/2, phospho-p38, p-38, phospho-JNK and JNK in cells treated with Qu and/or UVB. β-actin was used as loading control. B, C, D, E and F represent the densitometric analysis of B-raf, p-MEK/MEK, p-ERK/ERK, phospho-p38/p-38, phospho-JNK/JNK respectively. G, immunoblot analysis of PI3K-α and p-Akt in B16F10 cells at 24 h post-UVB and/ or Qu treatment. Signals were quantified for PI3K (H) and p-Akt (I) using Image Lab Software (Bio Rad). *, P<0.05; **, P<0.01 for control versus treatments; #, P<0.05, ##, P<0.01 for control versus UVB-alone treatment versus UVB + Qu treatments.

The mitogen-activated protein (MAP) kinase pathway, virtually activated in all melanomas, regulates cell proliferation, metastasis and survival [43]. We studied the effect of quercetin on MAPK signalling in UVB-irradiated B16F10 melanoma cells through western blotting. UVB irradiation of B16F10 cells decreased the expression of B-raf, phospho-MEK and, phospho-ERK. Treatment of UVB-irradiated B16F10 cells with quercetin induced a further decrease in the expression of phospho-MEK and its downstream effector phospho-ERK (Fig 8A–8D). Interestingly, phosphorylation of stress kinase p-38 increased upon treatment of UVB–irradiated B16F10 cells with quercetin (Fig 8A and 8E). However, quercetin caused no significant increase in UVB–induced phosphorylation of JNK (Fig 8A and 8F). Furthermore, treatment of UVB-irradiated B16F10 cells with quercetin markedly decreased the expression of PI3K and phosphorylation of its downstream effector Akt (Fig 8G–8I). Overall, these results suggest that combined UVB and quercetin treatment modulates mitogen-activated protein (MAP) kinase family proteins and attenuates PI3K/Akt survival signals in favour of increased apoptosis.

## Discussion

In this study, we investigated the molecular mechanism(s) by which quercetin, a bioflavonoid with limited toxicity on normal cells, is able to enhance UVB–induced cell death in B16F10 melanoma cells. In a therapeutic perspective, it is essential to specifically kill cancerous cells while sparing normal cells to avoid undesired toxicity. Therefore, we considered human HaCaT keratinocytes and Hs68 foreskin fibroblast cells as a reference model of non-tumorigenic skin cells. We found that quercetin (5–20 μM) is safe towards non-tumorigenic HaCaT and Hs68 cells, except for 40 μM quercetin which caused little cytotoxicity (Fig 1E and 1F). Unlike melanoma cells, quercetin caused no or minimal enhancement of UVB–induced cell death in non-tumorigenic Hs68 cells (Fig 1G, 1I and 1J). In melanoma therapy terms, these results are quite interesting considering that melanoma tumors and related cell lines are known to be exceptionally resistant to apoptosis [44]. The ability of quercetin to enhance the UVB–induced cell death in melanoma cells is an important observation because apoptosis is a mechanism of defence and acts by preventing the proliferation and dissemination of cancer cells into distant metastases.

The role of quercetin in chemoprevention of non-melanoma skin cancer has been investigated previously [19, 20]. In this study, it was found that quercetin, often referred as an antioxidant [19], actually, increased UVB-induced apoptotic response in B16F10 cells. Quercetin is a functionally pleiotropic kinase inhibitor, potentially capable of simultaneously inhibiting and modulating several intracellular signalling pathways. Due to its multiple intracellular targets, quercetin is capable of eliciting different responses and the net effect on cell fate is often the combination of multiple intracellular activities [45]. The pro-apoptotic effects of quercetin in UVB–irradiated B16F10 cells might be attributed to dissipation of mitochondrial membrane potential (Fig 4A), activation of caspases, cleavage of PARP-1 protein (Fig 2D), DNA damage (Fig 3D), and an increase in the percentage of sub-G1 apoptotic cells (Fig 3A and 3B). Together, these events constitute one of the mechanisms behind the efficacy of quercetin in UVB–irradiated B16F10 cells. Interestingly, 5–20 μM quercetin induced a dose dependent response in UVB–irradiated B16F10 cells, but differential response was observed at 40 μM quercetin and UVB co-treatment. The reason for the differences in the effects of quercetin on UVB–induced apoptotic response, especially the effect observed at 40 μM quercetin and UVB co-treatment, might be related to dose of quercetin administration, balance between the pro- and anti-oxidant activity of quercetin, anti-oxidant defence response of the cell and the influence of quercetin on multiple intracellular activities [45, 46].

In cells stimulated to die in response to UVB irradiation, reactive oxygen species (ROS) and intracellular Ca^2+^ changes are known to cause oxidative damage and finally cell death. It was observed that quercetin markedly increased the UVB–induced ROS generation in B16F10 cells (Fig 5A and 5B). Based on the results and observations in the present study, it may be suggested that quercetin could reduce the Nrf-2 expression and modulated the expression of downstream anti-oxidant enzymes (such as Cu-Zn SOD and catalase) that probably contributed to increased oxidative stress and DNA damage in UVB–irradiated B16F10 cells. Interestingly, blocking ROS formation using ascorbic acid as an anti-oxidant markedly reduced the pro-apoptotic effects of quercetin in UVB–irradiated B16F10 cells (Fig 5C). So far, this is the first report that establishes the pro-oxidant activity of quercetin as the key factor responsible for the growth inhibitory effects of quercetin in UVB–irradiated B16F10 cells. Further to these molecular events, quercetin increased the elevation of intracellular free Ca^2+^ in UVB–irradiated B16F10 cells (Fig 5D and 5F). Interestingly, addition of ascorbic acid could also reduce the elevation of intracellular Ca^2+^ ion levels in response to treatment of UVB–irradiated B16F10 cells with quercetin (Figs 6A and 5B). Hence, it may be suggested that quercetin accelerates UVB–induced ROS formation, leading to elevation of intracellular free Ca^2+^ levels and subsequently cell death. These results are in consistence with the literature citing the role of the excessive ROS and Ca^2+^ signals in mitochondrial membrane potential (ΔΨ_M_) depolarisation and activation of apoptotic and/or non-apoptotic cell death pathways (36, 37).

Further, reactive oxygen species play key role in the activation of NF-κB, a nuclear transcription factor that primarily resides in the cytoplasm of a cell and translocates to nucleus when activated [41, 47]. NF-κB is a major transcription factor and is known to induce the expression of several pro-apoptotic and anti-apoptotic proteins, depending upon the cellular context [15]. The ability of quercetin to enhance the UVB–induced NF-κB nuclear translocation (Fig 7E and 7G) is likely to interfere with the balance that exists between the positive and negative apoptotic regulators of Bcl-2 family. Over-expression of pro-survival Bcl-2 is common in many types of skin cancers and has been correlated with decreased sensitivity to radiation. Based on the results, it may be suggested that quercetin modulated the protein expression as well as altered the ratio of Bax to that of Bcl-2 in inducing cell death in UVB-irradiated B16F10 cells. Bim (bcl-2 interacting mediator of cell death), a highly pro-apoptotic BH3-only protein in Bcl-2 family, reveals outstanding pro-apoptotic potential in melanoma cells and strategies for its induction appear of interest [48]. The ability of quercetin to induce the expression of Bim together with the altered ratio of Bcl-2 to that of Bax is likely to contribute to inducing cell death in UVB–irradiated B16F10 cells.

Ultraviolet-B radiations are known to alter multiple intracellular signalling pathways in skin cells [49]. Classically, Ras-Raf-MEK-ERK signalling pathway plays a protective role [50]. Based on the results and observations, it could be suggested that quercetin decreased the activity of Raf-MEK-ERK signalling axis in favour of increased apoptosis (Fig 8A–8D). In response to UVB–irradiation, activation of p-38 mitogen activated protein kinase and c-Jun N-terminal kinase (JNK) is generally thought to induce apoptosis [16, 17]. Therefore, the activation of p-38 and JNK is likely to enhance the therapeutic efficacy of quercetin in UVB–irradiated B16F10 cells. The ability of quercetin to enhance the UVB–induced activation of p-38, albeit not that of JNK, is certainly in favour of increased apoptosis (Fig 8E and 8F). Further, quercetin disrupted phosphatidylinositol-3-kinase (PI3K)/Akt survival signalling pathway (Fig 8G–8I) which also corroborated with growth inhibitory effects of quercetin in UVB–irradiated B16F10 cells.

In conclusion, quercetin, often cited as an antioxidant, in fact, possesses the potential to be a potent UVB photosensitizer, which may be due to the sub-G1 cell cycle arrest, increase of cell apoptosis, disruption of NF-κB signalling and influence on the MAPKs and PI3K/Akt pathway. In melanoma therapeutic terms, these results are interesting because skin tumors are logically the potential target for treatment by external UVB irradiation, especially when tumors are multiple and surgically incurable but are easily accessible to external light exposure. Overall, the present data support the possibility of using quercetin and UVB irradiation on melanoma cells, which may have clinical relevance. Further mechanistic studies are required to confirm the therapeutic efficacy of quercetin in UVB–irradiated *in vivo* melanoma models.

## References

[1] Smalley KS. Understanding melanoma signaling networks as the basis for molecular targeted therapy. J Invest Dermatol. 2010;130(1):28–37. Epub 2009/07/03. 10.1038/jid.2009.177 . 19571822

[2] Siegel R, Naishadham D, Jemal A. Cancer statistics, 2013. CA Cancer J Clin. 2013;63(1):11–30. Epub 2013/01/22. 10.3322/caac.21166 . 23335087

[3] Lee CH, Wu SB, Hong CH, Yu HS, Wei YH. Molecular Mechanisms of UV-Induced Apoptosis and Its Effects on Skin Residential Cells: The Implication in UV-Based Phototherapy. Int J Mol Sci. 2013;14(3):6414–35. Epub 2013/03/23. 10.3390/ijms14036414 23519108 PMC3634415

[4] Clydesdale GJ, Dandie GW, Muller HK. Ultraviolet light induced injury: immunological and inflammatory effects. Immunol Cell Biol. 2001;79(6):547–68. Epub 2002/03/21. 10.1046/j.1440-1711.2001.01047.x . 11903614

[5] Davids LM, Kleemann B. Combating melanoma: the use of photodynamic therapy as a novel, adjuvant therapeutic tool. Cancer Treat Rev. 2011;37(6):465–75. Epub 2010/12/21. 10.1016/j.ctrv.2010.11.007 . 21168280

[6] Kulms D, Schwarz T. Molecular mechanisms of UV-induced apoptosis. Photodermatol Photoimmunol Photomed. 2000;16(5):195–201. Epub 2000/11/09. . 11068857 10.1034/j.1600-0781.2000.160501.x

[7] Ryu HC, Kim C, Kim JY, Chung JH, Kim JH. UVB radiation induces apoptosis in keratinocytes by activating a pathway linked to "BLT2-reactive oxygen species". J Invest Dermatol. 2010;130(4):1095–106. Epub 2010/01/22. 10.1038/jid.2009.436 . 20090768

[8] Beani JC. [Enhancement of endogenous antioxidant defenses: a promising strategy for prevention of skin cancers]. Bull Acad Natl Med. 2001;185(8):1507–25; discussion 26–7. Epub 2002/04/27. . 11974970

[9] Shindo Y, Witt E, Packer L. Antioxidant defense mechanisms in murine epidermis and dermis and their responses to ultraviolet light. J Invest Dermatol. 1993;100(3):260–5. Epub 1993/03/01. . 8440901 10.1111/1523-1747.ep12469048

[10] Hirota A, Kawachi Y, Itoh K, Nakamura Y, Xu X, Banno T, et al Ultraviolet A irradiation induces NF-E2-related factor 2 activation in dermal fibroblasts: protective role in UVA-induced apoptosis. J Invest Dermatol. 2005;124(4):825–32. Epub 2005/04/09. 10.1111/j.0022-202X.2005.23670.x . 15816842

[11] Lee JM, Li J, Johnson DA, Stein TD, Kraft AD, Calkins MJ, et al Nrf2, a multi-organ protector? FASEB J. 2005;19(9):1061–6. Epub 2005/06/30. 10.1096/fj.04-2591hyp . 15985529

[12] Danial NN. BCL-2 family proteins: critical checkpoints of apoptotic cell death. Clin Cancer Res. 2007;13(24):7254–63. Epub 2007/12/21. 10.1158/1078-0432.ccr-07-1598 . 18094405

[13] Vermeulen K, Berneman ZN, Van Bockstaele DR. Cell cycle and apoptosis. Cell Prolif. 2003;36(3):165–75. Epub 2003/06/20. . 12814432 10.1046/j.1365-2184.2003.00267.x PMC6496173

[14] Placzek M, Przybilla B, Kerkmann U, Gaube S, Gilbertz KP. Effect of ultraviolet (UV) A, UVB or ionizing radiation on the cell cycle of human melanoma cells. Br J Dermatol. 2007;156(5):843–7. Epub 2007/03/16. 10.1111/j.1365-2133.2007.07795.x . 17355234

[15] Ueda Y, Richmond A. NF-kappaB activation in melanoma. Pigment Cell Res. 2006;19(2):112–24. Epub 2006/03/10. 10.1111/j.1600-0749.2006.00304.x 16524427 PMC2668252

[16] Zarubin T, Han J. Activation and signaling of the p38 MAP kinase pathway. Cell Res. 2005;15(1):11–8. Epub 2005/02/03. 10.1038/sj.cr.7290257 . 15686620

[17] Dhanasekaran DN, Reddy EP. JNK signaling in apoptosis. Oncogene. 2008;27(48):6245–51. Epub 2008/10/22. 10.1038/onc.2008.301 18931691 PMC3063296

[18] Russo M, Palumbo R, Mupo A, Tosto M, Iacomino G, Scognamiglio A, et al Flavonoid quercetin sensitizes a CD95-resistant cell line to apoptosis by activating protein kinase Calpha. Oncogene. 2003;22(21):3330–42. Epub 2003/05/23. 10.1038/sj.onc.1206493 . 12761503

[19] Gibellini L, Pinti M, Nasi M, Montagna JP, De Biasi S, Roat E, et al Quercetin and cancer chemoprevention. Evid Based Complement Alternat Med. 2011;2011:591356 Epub 2011/07/28. 10.1093/ecam/neq053 21792362 PMC3136711

[20] Murakami A, Ashida H, Terao J. Multitargeted cancer prevention by quercetin. Cancer Lett. 2008;269(2):315–25. Epub 2008/05/10. 10.1016/j.canlet.2008.03.046 . 18467024

[21] Zhang W, Zhang F. Effects of quercetin on proliferation, apoptosis, adhesion and migration, and invasion of HeLa cells. Eur J Gynaecol Oncol. 2009;30(1):60–4. Epub 2009/03/26. . 19317259

[22] Aggarwal BB, Shishodia S. Molecular targets of dietary agents for prevention and therapy of cancer. Biochem Pharmacol. 2006;71(10):1397–421. Epub 2006/03/28. 10.1016/j.bcp.2006.02.009 . 16563357

[23] Cao HH, Tse AK, Kwan HY, Yu H, Cheng CY, Su T, et al Quercetin exerts anti-melanoma activities and inhibits STAT3 signaling. Biochem Pharmacol. 2014;87(3):424–34. Epub 2013/11/28. 10.1016/j.bcp.2013.11.008 . 24275163

[24] Menon LG, Kuttan R, Kuttan G. Inhibition of lung metastasis in mice induced by B16F10 melanoma cells by polyphenolic compounds. Cancer Lett. 1995;95(1–2):221–5. Epub 1995/08/16. . 7656234 10.1016/0304-3835(95)03887-3

[25] Rastogi RP, Richa, Kumar A, Tyagi MB, Sinha RP. Molecular mechanisms of ultraviolet radiation-induced DNA damage and repair. J Nucleic Acids. 2010;2010:592980 Epub 2011/01/07. 10.4061/2010/592980 21209706 PMC3010660

[26] Adil MD, Kaiser P, Satti NK, Zargar AM, Vishwakarma RA, Tasduq SA. Effect of Emblica officinalis (fruit) against UVB-induced photo-aging in human skin fibroblasts. J Ethnopharmacol. 2010;132(1):109–14. Epub 2010/08/07. 10.1016/j.jep.2010.07.047 . 20688142

[27] Olson ER, Melton T, Dong Z, Bowden GT. Stabilization of quercetin paradoxically reduces its proapoptotic effect on UVB-irradiated human keratinocytes. Cancer Prev Res (Phila). 2008;1(5):362–8. Epub 2009/01/14. 10.1158/1940-6207.capr-08-0101 19138980 PMC2609748

[28] Uchide N, Ohyama K, Bessho T, Toyoda H. Lactate dehydrogenase leakage as a marker for apoptotic cell degradation induced by influenza virus infection in human fetal membrane cells. Intervirology. 2009;52(3):164–73. Epub 2009/06/13. 10.1159/000224644 . 19521105

[29] Fishman D, Irena B, Kellman-Pressman S, Karas M, Segal S. The role of MHC class I glycoproteins in the regulation of induction of cell death in immunocytes by malignant melanoma cells. Proc Natl Acad Sci U S A. 2001;98(4):1740–4. Epub 2001/02/15. 10.1073/pnas.041591298 11172021 PMC29327

[30] Sarkar M, Varshney R, Chopra M, Sekhri T, Adhikari JS, Dwarakanath BS. Flow-cytometric analysis of reactive oxygen species in peripheral blood mononuclear cells of patients with thyroid dysfunction. Cytometry B Clin Cytom. 2006;70(1):20–3. Epub 2005/12/13. 10.1002/cyto.b.20082 . 16342062

[31] D'Anneo A, Carlisi D, Lauricella M, Emanuele S, Di Fiore R, Vento R, et al Parthenolide induces caspase-independent and AIF-mediated cell death in human osteosarcoma and melanoma cells. J Cell Physiol. 2013;228(5):952–67. Epub 2012/06/13. 10.1002/jcp.24131 . 22688575

[32] Rieber M, Strasberg Rieber M. Apoptosis-inducing levels of UV radiation and proteasome inhibitors produce opposite effects on p21(WAF1) in human melanoma cells. Int J Cancer. 2000;86(4):462–7. Epub 2000/05/08. . 10797256 10.1002/(sici)1097-0215(20000515)86:4<462::aid-ijc3>3.0.co;2-b

[33] Yu W, Sanders BG, Kline K. RRR-alpha-tocopheryl succinate-induced apoptosis of human breast cancer cells involves Bax translocation to mitochondria. Cancer Res. 2003;63(10):2483–91. Epub 2003/05/17. . 12750270

[34] Collins JA, Schandi CA, Young KK, Vesely J, Willingham MC. Major DNA fragmentation is a late event in apoptosis. J Histochem Cytochem. 1997;45(7):923–34. Epub 1997/07/01. . 9212818 10.1177/002215549704500702

[35] Cosentino K, Garcia-Saez AJ. Mitochondrial alterations in apoptosis. Chem Phys Lipids. 2014. Epub 2014/04/16. 10.1016/j.chemphyslip.2014.04.001 . 24732580

[36] Ly JD, Grubb DR, Lawen A. The mitochondrial membrane potential (deltapsi(m)) in apoptosis; an update. Apoptosis. 2003;8(2):115–28. Epub 2003/05/27. . 12766472 10.1023/a:1022945107762

[37] Bivik CA, Larsson PK, Kagedal KM, Rosdahl IK, Ollinger KM. UVA/B-induced apoptosis in human melanocytes involves translocation of cathepsins and Bcl-2 family members. J Invest Dermatol. 2006;126(5):1119–27. Epub 2006/03/11. 10.1038/sj.jid.5700124 . 16528366

[38] Salucci S, Burattini S, Battistelli M, Baldassarri V, Maltarello MC, Falcieri E. Ultraviolet B (UVB) Irradiation-Induced Apoptosis in Various Cell Lineages in Vitro. Int J Mol Sci. 2012;14(1):532–46. Epub 2012/12/29. 10.3390/ijms14010532 23271369 PMC3565280

[39] Masaki H, Izutsu Y, Yahagi S, Okano Y. Reactive oxygen species in HaCaT keratinocytes after UVB irradiation are triggered by intracellular Ca(2+) levels. J Investig Dermatol Symp Proc. 2009;14(1):50–2. Epub 2009/08/14. 10.1038/jidsymp.2009.12 . 19675553

[40] Morgan MJ, Liu ZG. Crosstalk of reactive oxygen species and NF-kappaB signaling. Cell Res. 2011;21(1):103–15. Epub 2010/12/29. 10.1038/cr.2010.178 21187859 PMC3193400

[41] van den Berg R, Haenen GR, van den Berg H, Bast A. Transcription factor NF-kappaB as a potential biomarker for oxidative stress. Br J Nutr. 2001;86 Suppl 1:S121–7. Epub 2001/08/25. . 11520430 10.1079/bjn2001340

[42] Lee DH, Cho KS, Park SG, Kim EK, Joo CK. Cellular death mediated by nuclear factor kappa B (NF-kappaB) translocation in cultured human lens epithelial cells after ultraviolet-B irradiation. J Cataract Refract Surg. 2005;31(3):614–9. Epub 2005/04/07. 10.1016/j.jcrs.2004.05.053 . 15811753

[43] Panka DJ, Atkins MB, Mier JW. Targeting the mitogen-activated protein kinase pathway in the treatment of malignant melanoma. Clin Cancer Res. 2006;12(7 Pt 2):2371s–5s. Epub 2006/04/13. 10.1158/1078-0432.ccr-05-2539 . 16609061

[44] Grossman D, Altieri DC. Drug resistance in melanoma: mechanisms, apoptosis, and new potential therapeutic targets. Cancer Metastasis Rev. 2001;20(1–2):3–11. Epub 2002/02/08. . 11831644 10.1023/a:1013123532723

[45] Russo GL, Russo M, Spagnuolo C, Tedesco I, Bilotto S, Iannitti R, et al Quercetin: a pleiotropic kinase inhibitor against cancer. Cancer Treat Res. 2014;159:185–205. Epub 2013/10/12. 10.1007/978-3-642-38007-5_11 . 24114481

[46] Ferraresi R, Troiano L, Roat E, Lugli E, Nemes E, Nasi M, et al Essential requirement of reduced glutathione (GSH) for the anti-oxidant effect of the flavonoid quercetin. Free Radic Res. 2005;39(11):1249–58. Epub 2005/11/22. 10.1080/10715760500306935 . 16298752

[47] Li N, Karin M. Is NF-kappaB the sensor of oxidative stress? FASEB J. 1999;13(10):1137–43. Epub 1999/07/01. . 10385605

[48] Plotz M, Gillissen B, Quast SA, Berger A, Daniel PT, Eberle J. The BH3-only protein Bim(L) overrides Bcl-2-mediated apoptosis resistance in melanoma cells. Cancer Lett. 2013;335(1):100–8. Epub 2013/02/14. 10.1016/j.canlet.2013.02.005 . 23402819

[49] Heck DE, Gerecke DR, Vetrano AM, Laskin JD. Solar ultraviolet radiation as a trigger of cell signal transduction. Toxicol Appl Pharmacol. 2004;195(3):288–97. Epub 2004/03/17. 10.1016/j.taap.2003.09.028 . 15020191

[50] Kitagawa D, Tanemura S, Ohata S, Shimizu N, Seo J, Nishitai G, et al Activation of extracellular signal-regulated kinase by ultraviolet is mediated through Src-dependent epidermal growth factor receptor phosphorylation. Its implication in an anti-apoptotic function. J Biol Chem. 2002;277(1):366–71. Epub 2001/11/06. 10.1074/jbc.M107110200 . 11694531

